# Antimicrobial Efficacy and Cytocompatibility of Calcium Hypochlorite Solution as a Root Canal Irrigant: An *in Vitro* Investigation

**DOI:** 10.7508/iej.2016.03.004

**Published:** 2016-05-01

**Authors:** Mahdi Sedigh-Shams, Ahmad Gholami, Abbas Abbaszadegan, Roohollah Yazdanparast, Milad Saberi Nejad, Azam Safari, Mohammadreza Nabavizadeh, Younes Ghasemi

**Affiliations:** a *Department of Endodontics, Dental School, Shiraz University of Medical Sciences, Shiraz, Iran; *; b * Pharmaceutical Sciences Research Center and Department of Pharmaceutical Biotechnology, Shiraz University of Medical Sciences, Shiraz, Iran; *; c * Students' Research Committee, Dental School, Shiraz University of Medical Sciences, Shiraz, Iran; *; d * Prevention of Oral and Dental Diseases Research Center, Dental School, Shiraz University of Medical Sciences, Shiraz, Iran*

**Keywords:** Calcium Hypochlorite, *Enterococcus faecalis*, Polymerase Chain Reaction, Root Canal Irrigant, Sodium Hypochlorite

## Abstract

**Introduction:**

The purpose of this study was to compare the antimicrobial efficacy of sodium hypochlorite (SH) and calcium hypochlorite (CH) against *Enterococcus faecalis* *(E. faecalis)* using quantitative real-time polymerase chain reaction (qPCR) analysis and also to compare their cytocompatibility on L929 murine fibroblasts using Mossman’s tetrazolium toxicity (MTT) assay.

**Methods and Materials:**

A broth micro-dilution susceptibility test was used to determine the minimum inhibitory concentration (MIC) of each irrigant against *E. faecalis*. Then, the root canals of 50 mature extracted human mandibular premolars were contaminated with *E. faecalis* and were randomly divided into three groups according to the irrigant used (*n*=20). Canals were irrigated with SH in group I (*n*=20) and CH in group II (*n*=20) at their obtained MIC. In group III (*n*=10), sterile saline was used. Microbial sampling was performed before and after biomechanical preparation. Quantitative PCR was used to quantify *E. faecalis* in the root canal samples. For cytocompatibility assessment, L929 murine fibroblasts were exposed to various concentrations of the irrigants.

**Results:**

Irrigation with test materials resulted in significant reduction in colony forming units (CFU) in post-instrumentation samples (with the MIC values of SH and CH against *E. faecalis* being 0.5% and 5%, respectively). However, the reduction in the normal saline group was not significant (*P*=0.203). In addition, 5% CH was more effective than 0.5% SH (*P*=0.006) in eliminating *E. faecalis*. Among the different concentrations of tested irrigants, 0.5% CH and 5% SH showed the least and the most cytotoxicity, respectively (*P*<0.001). The cytotoxicity of 5% CH and 0.5% SH was similar (*P*=0.99), and lower than 2.5% SH (*P*<0.001).

**Conclusion:**

CH at an MIC of 5% was effective in eliminating E. faecalis in planktonic state and also its biofilm and exhibited comparable cytocompatibility to that of 0.5% SH.

## Introduction

The major cause of apical periodontitis is microbial infection of the root canal system [1]. The host defense elements cannot easily reach the bacteria established in the root canal space; therefore, chemical irrigation together with mechanical instrumentation is crucial to achieve successful outcomes [2-4]. Using a biocompatible irrigation solution with an acceptable antimicrobial activity is crucial for complete chemomechanical preparation of the root canal space [1].

Among the several organisms residing in nonvital root canals, *Enterococcus faecalis* (*E. faecalis*) is considered as the most resistant to antimicrobial agents [5]. This pathogen can impede the action of endodontic irrigants because of its potential to invade root dentin and biofilm creation [5]. It can be stated that efforts to eliminate this bacterium may equate with achieving successful disinfection.

Among the various irrigants used, sodium hypochlorite (SH) has the advantage of excellent antibacterial properties and the ability to dissolve necrotic tissues [6, 7]. However, it is highly toxic to the living tissues especially at high concentrations and it cannot completely remove the bacteria and their bioburden from the root canal system [8]. Furthermore, it is stated that SH can produce dentinal defects during instrumentation [9]. Therefore, attempts continue to increase the antibacterial effectiveness of SH by adding proteolytic enzymes [10] and detergents [11] or by modifying its chemical structure and reactivity [12, 13].

SH and calcium hypochlorite (CH) belong to the same family of chemicals and they are primarily used as bleaching agents or disinfectants. In industry, CH has been traditionally used for disinfection and purification treatment of water and milk [14-16]. Unlike SH, it can be easily stored in powder form without losing stability [17]. The bactericidal action of these two agents is highly related to the level of available chlorine [6]. CH powder can produce more available chlorine when it comes in contact with water [18]. Furthermore, it can produce calcium hydroxide which in turn may enhance the antibacterial efficacy of the solution [18]. The antibacterial activity of these agents can also be relevant to their pH levels, which may affect the cytoplasmic membrane integrity, enzymatic action and cellular metabolism of the microorganisms [6].

In a preliminary study, no differences were found among the tissue dissolving properties of 5% or 10% CH and 4.65% SH (Chlorax solution) or 1.36% SH solutions after 60 min [18]. Further, it was revealed that similar to 10% SH, 10 and 15% CH could not produce any difference in microleakage when they were used as pretreatment agents prior to an acetone-based adhesive system [19]. In another study, Görduysus *et al.* [20] showed that nor SH either CH were effective in removing smear layer and dentinal debris.

There are limited investigations on antibacterial efficacy of CH in endodontic field [21, 22]. Recently, the antibacterial efficacy of 2.5% CH and 2.5% SH in elimination of *E. faecalis* from the contaminated root canals were investigated. It was found that CH had better antibacterial activity than SH when agitated with ultrasonic energy [21]. In another study, Schmidt *et al.* [22] indicated that 2.5% CH can effectively be used for decontamination of gutta-percha points. 

To the best of our knowledge, there is no study that have assessed the optimum concentration for antibacterial activity and cytocompatibility of CH in comparison with SH. The purpose of this *in vitro* study was to compare the antimicrobial efficacy of CH and SH at their minimum inhibitory concentrations (MIC) in root canals contaminated with *E. faecalis* using quantitative polymerase chain reaction (qPCR) and to assess their cytocompatibility with different concentrations on L929 murine fibroblasts.

## Materials and Methods

The irrigants used in this study were 5% SH (Sigma-Aldrich Co., St. Louis, MO, USA) which was diluted from its initial concentration and 10% CH (Star-Chlon, Japan) (CH powder was mixed with double-distilled water using magnetic stirrer) and sterile saline. The pH of each solution was assessed using an electronic pH meter (Labtron PHT 16, Tehran, Iran).

***Determination of minimum inhibitory concentration (MIC)***

MIC values of CH and SH were evaluated according to the following method. A serial two-fold dilution of each solution was prepared in a 96-well microplate using 90 µL of brain heart infusion (BHI) broth medium (Himedia Laboratories, Mumbai, India). *E. faecalis* (ATCC 29212) was cultured in BHI broth and adjusted for the turbidity equivalent to 0.12 at an optical density (OD) of 600 that resulted in 1-1.5×10^8^ colony forming units per milliliters (CFU/mL) bacteria. This inoculum was then diluted to yield 5×10^6^ CFU/mL. Finally, 10 µL of the prepared inoculum suspensions was added to the microplate and incubated at 37^°^C and 5% CO_2_. After 24 h, the OD of the wells was obtained by a plate reader (BioTek, PowerWave XS2, Vermont, USA) apparatus at 600 OD. Culture media were used as the negative control group. The lowest concentration of each irrigant that inhibits 90% of *E. faecalis* growth in comparison to the negative control (after an overnight incubation) was defined as the MIC value. All experiments were performed in triplicates.

***Sample collection and preparation***

This study was approved by the Research Ethics Committee of Shiraz University of Medical Sciences, Shiraz, Iran (Grant No.: 6004). A total of 50 intact extracted human mandibular premolars with single canals and without any resorption or cracks were selected. The surface of the teeth were scrapped to clean the soft tissue residues and disinfected by 2.5% SH for 20 min. Then the teeth were stored in sterile saline until use.

After access cavity preparation, working length (WL) was determined by subtracting 1 mm from the length of a #10 K-file (Mani, Tochigi, Japan) after its emergence from the apical foramen. To standardize the diameter of apical constriction, each root canal was enlarged with a #20 K-file. Each tooth was placed in a glass tube separately and sterilized in an autoclave at 121^°^C for 30 min. To evaluate the efficiency of sterilization process, 10 samples were immersed completely in 10 mL autoclaved BHI broth and kept in an incubator at 37^°^C for 96 h and then checked for turbidity.

The assembly of the samples was performed according to the methodology suggested by Camara *et al.* [23]. A hole was generated in the center of the rubber stopper of the glass vials and each tooth was inserted into the generated hole with pressure until its root set firmly inside the vial. Then, the interface of the teeth and rubber stopper was thoroughly sealed with cyanoacrylate glue (Razi Institute, Karaj, Iran). The seal of the assembly was carefully checked and ensured using 0.5% methylene blue dye; if the dye could not reach the vial under luminal air flow chamber, the seal was confirmed.

Each assembly was then autoclaved and filled with BHI broth under a laminar air flow chamber and stored in an incubator at 37^°^C for 96 h. The sterility of the samples was ensured when no turbidity was detected.

***Root canal contamination***

Colonies of pure cultures of *E. faecalis* (ATCC 29212) on BHI agar plates were supplemented with 10% sheep blood and suspended in a sterile saline solution. Spectrophotometric evaluation was used to confirm that the number of bacteria reached 3×10^8^ CFU/mL (equal to 1.0 McFarland standard).

All experimental procedures were performed under strict sterile conditions under the laminar air flow chamber. Each root canal was contaminated with 10 µL of the bacterial suspension by inserting sampler microtip (Eppendorf, Hamburg, Germany) into the access cavities. Then, the bacterial suspension was introduced into the whole length of the canal using a #20 K-file up to the WL with a gentle pumping motion. If the turbidity of the medium samples reached 12×10^8^ CFU/mL (equal to 4 McFarland) during the incubation period for all the samples, the positive bacterial growth was confirmed. The specimens were stored in an incubator at 37^°^C and 95% relative humidity for 21 days. The purity of *E. faecalis* was identified by gram staining and microscopic observation of the colony morphology.

***Microbial sampling***

The teeth were randomly divided into three groups (20 specimens in each experimental group and 10 specimens in the control group). In groups I and II, SH and CH were used as root canal irrigants, respectively, each at their obtained MIC in the previous phase. The initial microbial assessment (S1) was carried out after the incubation period using three sterile #20 paper points (Gapadent Co Ltd, Tianjin, China) that were kept in each canal for 1 min. In short, each root canal was filled with 20 µL sterile saline solution and a #20 K-file was inserted into the root canals to reach the WL with a gentle filling motion. To detach the microorganisms from the inner root surfaces, an ultrasonic tip was placed in contact with the file shank and activated for 1 min; then, the root canals were sampled. The paper points were transferred into a dry tube containing BHI media under aseptic conditions and frosted immediately at -20^°^C. After this stage, the contaminated vials were exchanged with the empty sterile ones.

The root canals were instrumented with ProTaper rotary instruments (S1, S2, F1, F2, F3) (Dentsply, Maillefer, Ballaigues, Switzerland) set on an electric motor (Endo IT Professional, Aseptico Inc., WA, USA) according to the manufacturer’s instructions. The irrigants were freshly prepared (at their MICs) just before starting the experiment for the assigned experimental groups. Between the instruments, 2 mL of each solution was used (a total of 10 mL for each root canal). The irrigation was performed using disposable syringes with a 30-gauge needles.

After complete chemomechanical preparation, the samples in groups I and II were irrigated with 5 mL of 5% sodium thiosulfate as a neutralizer and then rinsed with 5 mL sterile saline. Final sampling (S2) was performed using a similar method for initial sampling.

***Quantitative real-time PCR***

Microbiological evaluation of the samples was performed using real-time PCR. The genomic DNA was isolated using a bacterial genomic DNA isolation kit (Promega, Madison, WI, USA) according to the manufacturer’s suggested protocol but scaled down to accommodate 100 samples. The concentration and purity of the DNA samples were determined using a UV-VIS spectrophotometer. The pure DNA obtained from *E. faecalis* was decimally diluted in a sterile Tris-EDTA buffer (TE) with pH value of 8.0, and stored at -20^°^C [24].

The primers were designed by allele ID software (Premier Biosoft International, Palo Alto, CA, USA) to amplify a 138-base pair fragment of the gelatinase (*gelE*) gene as a target sequence. The sequences of forward and reverse primers for the *E. faecalis* *gelE* gene were as follows: F(5′ ACA CCA TTA TCC AGA ACT TAG GC 3′) and R(5′ GCT GCT GAC ACC ACT GAA G 3′). The specificity of the primer sequences was tested by homology searches in the nucleotide database [NCBI, nucleotide BLAST (BLASTN)] [25].

Negative PCR controls (no DNA template) and negative qPCR amplification controls (*E. faecalis *total DNA template) were prepared in duplicate alongside experimental total DNA sample to normalize any background signal obtained from the following amplification. To estimate the number of *E. faecalis *and the total number of bacteria in the samples, DNA from* E. faecalis* was purified within a concentration range of 10^1^ to 10^6^ genomic DNA copies per reaction and then the linearity and efficiency of the PCR assay were determined. Each sample dilution (1 µL) was added to five replicas in PCR tubes and the real-time PCR machine was started. The efficiency of the *gelE*-based real-time PCR assay was calculated by the following formula: Efficiency=[10^(−1/slope)^]-1. 

Real-time PCR and data analysis were performed in iCycler iQ real-time detection system (Bio-Rad Laboratories, Hercules, CA, USA) using 2×SYBR premix Ex TaqTM II (Takara, Tokyo, Japan). The PCR conditions were as follows: 95^°^C for 5 min, then 50 cycles consisting of 95^°^C for 15 sec, and 60^°^C for 1 min. The melting curve (Tm) analysis of the final PCR product was performed from 50 to 90^°^C at 1^°^C intervals. An automatic threshold setting of 0.2 Nano grams was used for all samples. A positive reaction and a non-template reaction were included in all experiments.

The standard curves were constructed from a dilution series with a concentration range from 2×10^6^ to 2 CFU/mL of the sample. The DNA was extracted, quantified, and amplified as described above and then the standard curves were constructed. The correlation coefficient (R^2^) and the efficiency of the amplification were calculated [24, 26].

***Cytotoxicity assessment***

In order to compare the cytotoxicity of 0.5% and 5% CH and 0.5%, 2.5%, and 5% SH in L929 murine fibroblasts, an MTT colorimetric assay was used. Aliquots of 100 μL solution of RPMI 1640 medium comprising 1×10^4^ cells of L929 fibroblasts were placed in the wells of a 96-well cell culture plate and incubated at 37^°^C in the presence of 5% CO_2_ and 95% air for 24 h. Then, 100 μL of RPMI medium containing different concentrations of each sample was replaced with media in each well. The medium was discarded after 24 h of incubation and the wells were washed twice with 20 μL of fresh medium for 2-3 min. For staining the viable cells, 25 μL of MTT [3-(4,5 dimethylthiazole-2-yl)-2,5-diphenyl tetrazolium] solution (Sigma-Aldrich Co., St. Louis, MO,USA) was added to each well and incubated in a humidified atmosphere for 3 h. Then, 100 μL of DMSO was added to each well and the culture plate was vibrated for 10 min. ELISA microplate reader (ELX808 absorbance microplate reader; BioTek Instruments Inc., Winooski, VT, USA) was used to determine the light absorbance of the experimental samples, culture medium (negative control), and 35% H_2_O_2_ (positive control). All tests were performed in three replicates and the absorbance values were read three times. The percentage of the mean optical density (OD) values of the experimental solutions relative to the OD value of the negative control was expressed as the mean cell viability.

***Statistical analysis***

The Kruskal-Wallis and Mann-Whitney U tests were used to compare the CFU counts in S1 or S2 samples among the groups. The Wilcoxon signed-ranks test was also used to compare the CFU counts before and after chemomechanical preparations in each group. One-way analysis of variance (ANOVA) and Tukey’s tests were used to evaluate the differences in the mean cell viability values. The level of significance was set at 0.05.

## Results

The MIC values for SH and CH against *E. faecalis* were 0.5% and 5%, respectively. The pH values of the experimental solutions were 11.57, 11.98, and 7.39 for 5% SH, 0.5% CH and 0.9% sterile saline, respectively.

Regarding the CFU counts, the Wilcoxon signed-rank test showed a significant difference between the S1 and S2 samples in groups I and II (*P*<0.001), but no difference was observed in control group (*P*=0.203). Table 1 shows the median (mean+SD) CFU counts for S1 and S2 samples in each group. Statistical analyses revealed no significant differences among the groups in S1 samples (*P*=0.768), but the difference was significant among the groups in S2 samples (*P*<0.001). Moreover, 5% CH and 0.5% SH solutions were more efficient than normal saline in reducing the CFU counts in S2 samples (*P*<0.001). It was showed that 5% CH was significantly more effective than 0.5% SH in CFU reduction (*P*=0.006).

The 35% H_2_O_2_ solution resulted in 99.82% cell death. One-way ANOVA test revealed a significant difference between the experimental groups (*P*<0.001). Tukey’s post hoc test showed 0.5% CH and 5% SH to be the least and the most cytotoxic solutions (*P*<0.001). Furthermore, 5% CH and 0.5% SH had similar cytotoxicity (*P*=0.99) which was less than that of 2.5% SH (*P*<0.001). The results are summarized in Table 2.

## Discussion

Despite the advances in the techniques employed in root canal treatment, more than 35% of the root canal surfaces may be left untouched by the instruments during root canal preparation [1]. Therefore, using antimicrobial disinfectants with adequate efficacy against endodontic microorganisms is critical for complete preparation of the root canal system [2, 27]. In limited investigations performed on antimicrobial activity of CH in the field of dentistry, this material showed promising results. de Almeida *et al. *[21] found that CH had better antibacterial action in comparison with SH as a root canal irrigant after agitating with ultrasonic energy. In another investigation, Twomey *et al.* [28] tested the efficacy of CH for disinfection of dental stones. They revealed that when the dental stones were mixed with water containing 0.5% CH, the number of residual microorganisms reduced. Also, there are several reports on antimicrobial efficacy of this material in industry [14-17, 29]. 

Availability of more chlorine in CH solution compared to SH predicts a promising antimicrobial activity of this material for endodontic purposes; however its cytotoxicity should also be considered. This solution can be prepared in different concentrations by mixing the powder of CH with distilled water. Compared to SH, CH is rather more stable and easily storable [15]. Therefore, closer assessment of this material as an endodontic disinfectant seems rational.

**Table 1:** Median (Mean±SD) of colony forming unit (CFU) counts in pre- (S1) and post- (S2) instrumentation samples

**Irrigation solutions**	**S1 CFU**	**S2 CFU**
**5% calcium hypochlorite**	2.28 × 10^9 ^(6.56±8.76 × 10^9^) ^A,a^	5.44 × 10^2 ^(3.02±9.80 × 10^5^) ^B,a^
**0.5% sodium hypochlorite**	1.97 × 10^9 ^(4.66±6.85 × 10^9^) ^A,a^	4.35 × 10^3 ^(4.41±1.87 × 10^7^) ^B,b^
**Sterile saline**	2.09 × 10^9 ^(4.49±4.29 × 10^9^) ^A,a^	1.72 × 10^9 ^(2.04±2.03 × 10^9^) ^A,c^

*Horizontally, uppercase letters denote pre-instrumentation and post-instrumentation CFU comparisons in each group*

*Vertically, lowercase letters denote comparisons between groups. Equal letters denotes a lack of statistically significant difference*

**Table 2:** Mean (SD) of viability of fibroblasts after treatment with different concentrations of test irrigants

**Groups**	**Cell viability **
**0.5% ** **calcium hypochlorite**	100.16±1.72 ^a^
**5% calcium hypochlorite**	69.10±8.10 ^b^
**0.5% ** **sodium hypochlorite**	70.99±6.34 ^b^
**2.5% ** **sodium hypochlorite**	31.19±4.47 ^c^
**5% ** **sodium hypochlorite**	10.69±4.24 ^d^

*Having a letter in common denotes the lack of statistically significant difference*

The present study was initiated based on the acceptable results of a previous work on tissue-dissolving capacity of this material compared to SH [18]. After 60 min of exposure, no differences was observed regarding the tissue dissolving properties of 5% and 10% CH compared to 4.65% or 1.36% SH solutions. Later on, de Almeida e*t al. *[21] compared the antibacterial efficacy of 2.5% SH and 2.5% CH solutions associated with passive ultrasonic irrigation in root canals infected with *E. faecalis*. They found no significant antibacterial differences between these two irrigants when no ultrasonic agitation was applied. However the authors did not present any reason selecting 2.5% concentration of this material. Moreover, no previous report was present in literature regarding its level of cytotoxicity. Thus, in the first phase of our experiment we determined the MICs of our experimental solutions to select their optimal concentration to be used in the root canals.

It was found that CH solution at its MIC concentration was more efficient than 0.5% SH in elimination of planktonic and biofilm forms of *E. faecalis* and exhibited comparable cytocompatibility to that of 0.5% SH. It was also showed that the pH value of 5% CH was more than 0.5% SH and this might be a reason for its higher antibacterial activity. A previous investigation also revealed that the alkaline property of alkaline solutions of hypochlorite can improve the bactericidal efficacy against the anaerobic bacteria [30]. In addition, CH granules partially dissolve in an aqueous solution and liberate both hypochlorous acid and CH and result in an increased pH level [31]. It also has been found that exposure of *E. faecalis *to CH at pH 11.1 for 30 min results in 0.4% cell survival and a relatively small increase in alkalinity (pH 11.5) which can *per se *increase the level of antibacterial activity [32]. Furthermore, CH can dissolve pulp tissue remnants and this synergism is in favorite of canal disinfection in inaccessible areas such as fins and isthmuses [18].

For contamination of the root canals, the methodology presented by Camara *et al.* [23] was employed. The advantage of this system was to provide nutrition for instant bacterial growth during the formation of biofilm as the periodontium do for intracanal bacteria in clinical situations.

In the present investigation, a culture-independent molecular microbiology method was employed. The advantage of this method is its higher sensitivity which potentially targets culturable viable cells and viable but nonculturable cells [33]. Also qPCR approach allows the quantification of individual target species as well as total bacteria in clinical samples. Another advantage of this technique is the limited contamination due to avoidance of post amplification manipulation. Furthermore, bacterial numbers could be estimated contrary to culturing methods [33]. However, in this method, detection of free floating DNAs and RNAs from nonviable cells may cause overestimated results. [33]. 

Although antimicrobial effectiveness is the most important property required for an irrigation solution [34, 35], the biological considerations in case of inadvertently extrusion into periapical tissues should also be considered [36]. As a result, the cytocompability assessment is also included in this study. To the best of our knowledge there is no study on cytotoxicity of CH on the normal cell-lines. In the present study, an immortalized L929 cell line was employed to compare the cytotoxicity of the experimental medicaments. This cell line was adopted because it is a well-characterized cell model and has been previously employed to determine the cytotoxicity of root canal irrigants [37, 38]. The cytotoxicity of dental material is usually evaluated by the MTT assay as an accurate index. The main advantage of this technique is its reproducibility. In addition, it does not need a washing step that causes some alternations in the sample [39]. The findings of current study showed that 0.5% CH was completely cytocompatible to L929 cell line; however, the concentration of 0.5% was lower than the MIC achieved for this substance. The 5% concentration of CH showed better antibacterial activity than 0.5% SH with no increase in cytotoxicity. This result might be related to the possible slower release of chlorine in CH compared to SH.

It should be emphasized that, as with most *in vitro* studies, the findings of the current study remain to be clinically confirmed. Further studies on other microorganisms other than *E. faecalis* can also be helpful to justify the antimicrobial activity of CH. Besides, studies on biocompatibility of this material in animal models can be recommended for future investigations.

## Conclusion

The present study provided new data on biological properties of calcium hypochlorite as an endodontic irrigant. The results of this study indicated that 5% calcium hypochlorite had similar cytotoxicity to 0.5% sodium hypochlorite and was more effective than NaOCl in eliminating *E. faecalis* biofilms.

## References

[1] Haapasalo M, Endal U, Zandi H, Coil JM (2005). Eradication of endodontic infection by instrumentation and irrigation solutions. Endod Topics.

[2] Byström A, Sundqvist G (1981). Bacteriologic evaluation of the efficacy of mechanical root canal instrumentation in endodontic therapy. Eur J Oral Sci.

[3] Dalton BC, Ørstavik D, Phillips C, Pettiette M, Trope M (1998). Bacterial reduction with nickel-titanium rotary instrumentation. J Endod.

[4] Peters O, Schönenberger K, Laib A (2001). Effects of four Ni–Ti preparation techniques on root canal geometry assessed by micro computed tomography. Int Endod J.

[5] Stuart CH, Schwartz SA, Beeson TJ, Owatz CB (2006). Enterococcus faecalis: its role in root canal treatment failure and current concepts in retreatment. J Endod.

[6] Estrela C, Estrela CR, Barbin EL, Spanó JCE, Marchesan MA, Pécora JD (2002). Mechanism of action of sodium hypochlorite. Braz Dent J.

[7] Rahimi S, Janani M, Lotfi M, Shahi S, Aghbali A, Vahid Pakdel M, Salem Milani A, Ghasemi N (2014). A review of antibacterial agents in endodontic treatment. Iran Endod J.

[8] Rôças IN, Siqueira JF (2011). Comparison of the in vivo antimicrobial effectiveness of sodium hypochlorite and chlorhexidine used as root canal irrigants: a molecular microbiology study. J Endod.

[9] Adl A, Sedigh-Shams M, Majd M (2015). The effect of using RC prep during root canal preparation on the incidence of dentinal defects. J Endod.

[10] Poggio C, Arciola CR, Dagna A, Chiesa M, Sforza D, Visai L (2010). Antimicrobial activity of sodium hypochlorite-based irrigating solutions. Int J Artif Organs.

[11] Wang Z, Shen Y, Ma J, Haapasalo M (2012). The effect of detergents on the antibacterial activity of disinfecting solutions in dentin. J Endod.

[12] Sirtes G, Waltimo T, Schaetzle M, Zehnder M (2005). The effects of temperature on sodium hypochlorite short-term stability, pulp dissolution capacity, and antimicrobial efficacy. J Endod.

[13] Wyckoff E, Natalie D, Nolan JM, Lee M, Hsieh T (1989). Structure of the Drosophila DNA topoisomerase II gene. Nucleotide sequence and homology among topoisomerases II. J Mol Biol.

[14] Peñas E, Gómez R, Frías J, Vidal-Valverde C (2010). Effects of combined treatments of high pressure, temperature and antimicrobial products on germination of mung bean seeds and microbial quality of sprouts. Food control.

[15] Wilson JK (1915). Calcium hypochlorite as a seed sterilizer. Am J Bot.

[16] Rutala WA, Weber DJ (1997). Uses of inorganic hypochlorite (bleach) in health-care facilities. Clin Microbiol Rev.

[17] Nwaukwa SO, Keehn PM (1982). The oxidation of alcohols and ethers using calcium hypochlorite [Ca (OCl) 2]. Tetrahedron Lett.

[18] Dutta A, Saunders WP (2012). Comparative evaluation of calcium hypochlorite and sodium hypochlorite on soft-tissue dissolution. J Endod.

[19] Bortoluzzi de Conto Ferreira M, Carlini Júnior B, Galafassi D, Gobbi DL (2015). Calcium hypochlorite as a dentin deproteinization agent: Microleakage, scanning electron microscopy and elemental analysis. Microsc Res Tech.

[20] Görduysus M, Küçükkaya S, Bayramgil NP, Görduysus MÖ (2015). Evaluation of the effects of two novel irrigants on intraradicular dentine erosion, debris and smear layer removal. Restor Dent Endod.

[21] de Almeida AP, Souza MA, Miyagaki DC, Dal Bello Y, Cecchin D, Farina AP (2014). Comparative evaluation of calcium hypochlorite and sodium hypochlorite associated with passive ultrasonic irrigation on antimicrobial activity of a root canal system infected with Enterococcus faecalis: An in vitro study. J Endod.

[22] Schmidt MHM, Sallenave RF, Demarchi A, Farina AP, Cecchin D, Souza MA (2015). Effectiveness of different auxiliary chemical substances in the rapid disinfection of gutta-percha points-an in vitro study. Rev Fac Odontol.

[23] Câmara AC, de Albuquerque MM, Aguiar CM, de Barros Correia ACR (2009). In vitro antimicrobial activity of 0.5%, 1%, and 2.5% sodium hypochlorite in root canals instrumented with the ProTaper Universal system. Oral Surg Oral Med Oral Pathol Oral Radiol Endod.

[24] Kumar R, Surendran P, Thampuran N (2010). Rapid quantification of Salmonella in seafood by real-time PCR assay. J Microbiol Biotechnol.

[25] Malorny B, Bunge C, Helmuth R (2007). A real-time PCR for the detection of Salmonella Enteritidis in poultry meat and consumption eggs. J Microbiol Methods.

[26] Nam H-M, Srinivasan V, Gillespie BE, Murinda SE, Oliver SP (2005). Application of SYBR green real-time PCR assay for specific detection of Salmonella spp. in dairy farm environmental samples. Int J Food Microbiol.

[27] Mohammadi Z, Shalavi S, Giardino L, Palazzi F, Asgary S (2015). Impact of Ultrasonic Activation on the Effectiveness of Sodium Hypochlorite: A Review. Iran Endod J.

[28] Twomey JO, Abdelaziz KM, Combe EC, Anderson DL (2003). Calcium hypochlorite as a disinfecting additive for dental stone. J Prosthet Dent.

[29] Buchholz A, Matthews K (2010). Reduction of Salmonella on alfalfa seeds using peroxyacetic acid and a commercial seed washer is as effective as treatment with 20 000 ppm of Ca (OCl) 2. Lett Appl Microbiol.

[30] Fischer R, Huerta J (1984). Effects of pH on microbial flora of necrotic root canals. J Endod.

[31] Law A, Messer H (2004). An evidence-based analysis of the antibacterial effectiveness of intracanal medicaments. J Endod.

[32] Siqueira J, Lopes H (1999). Mechanisms of antimicrobial activity of calcium hydroxide: a critical review. Int Endod J.

[33] Sedgley C, Nagel A, Dahlén G, Reit C, Molander A (2006). Real-time quantitative polymerase chain reaction and culture analyses of Enterococcus faecalis in root canals. J Endod.

[34] Gomes BP, Martinho FC, Vianna ME (2009). Comparison of 2.5% sodium hypochlorite and 2% chlorhexidine gel on oral bacterial lipopolysaccharide reduction from primarily infected root canals. Journal of Endodontics.

[35] Martinho FC, Chiesa WM, Zaia AA, Ferraz CC, Almeida JF, Souza-Filho FJ, Gomes BP (2011). Comparison of endotoxin levels in previous studies on primary endodontic infections. J Endod.

[36] Zhang W, Torabinejad M, Li Y (2003). Evaluation of cytotoxicity of MTAD using the MTT-tetrazolium method. J Endod.

[37] Abbaszadegan A, Gholami A, Mirhadi H, Saliminasab M, Kazemi A, Moein MR (2015). Antimicrobial and cytotoxic activity of Ferula gummosa plant essential oil compared to NaOCl and CHX: a preliminary in vitro study. Restor Dent Endod.

[38] Abbaszadegan A, Nabavizadeh M, Gholami A, Aleyasin Z, Dorostkar S, Saliminasab M, Ghasemi Y, Hemmateenejad B, Sharghi H (2015). Positively charged imidazolium‐based ionic liquid‐protected silver nanoparticles: a promising disinfectant in root canal treatment. Int Endod J.

[39] Mosmann T (1983). Rapid colorimetric assay for cellular growth and survival: application to proliferation and cytotoxicity assays. J Immunol Methods.

